# Neuroinflammation and Mental Health in Multiple Sclerosis and Autoimmune Encephalitis: Bridging Biological Mechanisms and Psychosocial Factors

**DOI:** 10.26502/aimr.0240

**Published:** 2026

**Authors:** Rojawn Khoshnam, Nika Khosravi Kia, Devendra K. Agrawal

**Affiliations:** 1 Department of Translational Research, College of Osteopathic Medicine of the Pacific, Western University of Health Sciences, Pomona, California 91766 USA

**Keywords:** Autoimmune encephalitis, Biopsychosocial model, Cognitive impairment, Immunotherapy, Multiple sclerosis, Neurodegeneration, Neuroimmunology, Neuroinflammation

## Abstract

Autoimmune neuroinflammatory diseases, including multiple sclerosis (MS) and autoimmune encephalitis (AE), are characterized by dysregulated immune activity within the central nervous system that leads to both neurological and psychiatric symptoms. While MS is typically a chronic condition indicated by demyelination and progressive neurodegeneration, AE often presents more acutely through antibody-mediated disruption of synaptic function. Despite differences in disease course and mechanisms, both conditions highlight the significant impact of immune-mediated processes on cognition, mood, and behavior. Current treatment options primarily focus on reducing inflammation and controlling disease activity through immunomodulatory or immunosuppressive therapies. Although these approaches have improved neurological outcomes, many patients continue to experience persistent cognitive impairment and psychiatric symptoms, including depression, anxiety, and executive dysfunction. These symptoms are increasingly recognized as core features of disease rather than secondary symptoms. New research suggests that immune system activity works together with brain network changes and chronic stress to shape mental and neurological symptoms. However, these areas are often overlooked in clinical care and understudied in research. Future directions should prioritize integrating cognitive and psychiatric outcomes into clinical trials, developing biomarkers that link immune activity to neuropsychiatric symptoms, and implementing multidisciplinary treatment approaches. A more comprehensive understanding of both biological and psychosocial contributors is essential for improving long-term patient outcomes and quality of life.

## Introduction

Autoimmune neuroinflammatory diseases are a broad group of diseases in which dysregulated immune activity within the central nervous system (CNS) leads to inflammation, neuronal injury, and clinical dysfunction [1]. Key cytokines and immune pathways have been implicated in the pathogenesis of the neuroinflammatory disease by promoting immune cell recruitment and maintaining inflammatory states within the CNS [1–8].

Multiple sclerosis (MS) is a chronic neuroinflammatory autoimmune disease that is steadily affecting more of the population. Demyelination of neurons in the CNS from an autoreactive T-helper cell response leads to inflammation, axonal injury, and impaired saltatory conduction, which underlies the progressive neurological deficits seen in multiple sclerosis [9–11]. Additionally, psychiatric disorders are more prevalent amongst people with MS than the general population [12].

Autoimmune encephalitis (AE) is an (often) acute neuroinflammatory autoimmune disorder that is also increasing in incidence [13]. In many cases, AE is driven by neuronal autoantibodies targeting extracellular antigens, including NMDAR and LGI1, leading to synaptic receptor dysfunction and subsequent neurological symptoms. However, some cases occur through antibodies against intracellular antigens, often in combination with malignancy [13]. Clinical manifestations of autoimmune encephalitis frequently mimic primary psychiatric disorders, complicating diagnosis, especially in individuals with preexisting behavioral or psychiatric conditions [14].

While autoimmune encephalitis is treated acutely with immunosuppressive therapies such as high-dose corticosteroids, IVIG, or plasmapheresis, multiple sclerosis management primarily relies on long-term disease-modifying therapies to prevent relapse and disease progression, with corticosteroids reserved for acute exacerbations [15,16]. Depending on symptom severity for AE, tumor treatment can sometimes be necessary as well.

Although immunomodulatory therapies address the underlying inflammatory process in autoimmune encephalitis and multiple sclerosis, cognitive and psychiatric symptoms frequently persist and require adjunctive, symptom-directed interventions [6]. These include pharmacologic management of mood, psychosis, and behavioral disturbances, as well as nonpharmacologic approaches such as cognitive rehabilitation and psychotherapy to optimize functional recovery and quality of life [15,17]. This review seeks to highlight current treatment successes while identifying gaps in the management of cognitive and psychiatric symptoms in autoimmune encephalitis and multiple sclerosis.

## Multiple Sclerosis: An Overview, underlying Mechanisms and Psychiatric Manifestations

**Figure 1:**
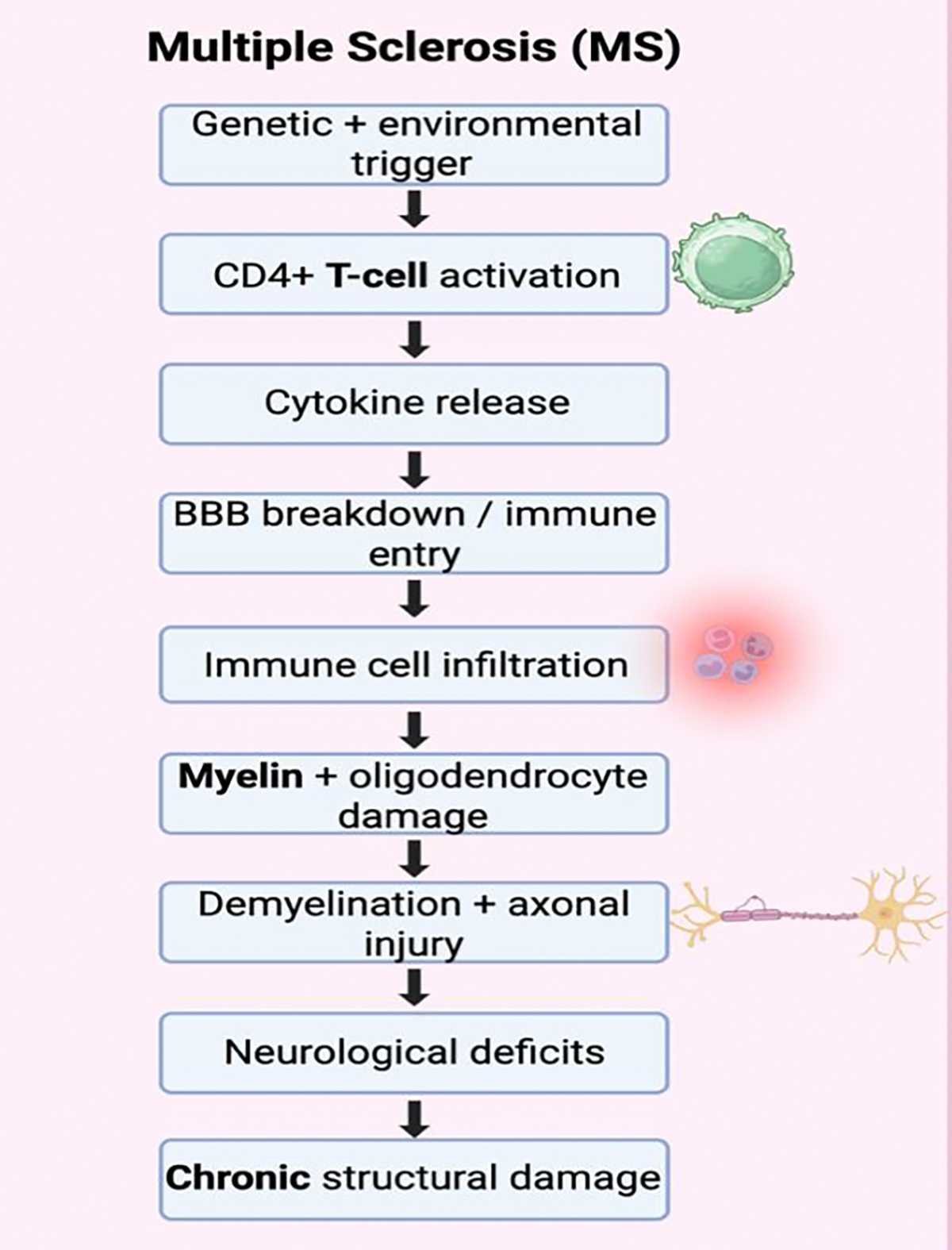
Pathophysiological Mechanisms of Multiple Sclerosis. Genetic factors and environmental trigger activate CD4+ T cells to release inflammatory cytokines that breakdown the blood-brain barrier (BBB), induce immune cell infiltration in the brain leading to injury and damage to various brain cells, demyelination and other chronic structural damage. This results in cognitive/psychiatric deficits.

Multiple sclerosis is characterized by inflammatory demyelinating plaques and gliotic scarring within the central nervous system, accompanied by axonal injury [18]. The prevailing pathogenic mechanism involves autoreactive CD4^+^ proinflammatory T cells, particularly Th1 and Th17 subsets. These immune cells cross the blood–brain barrier, initiating a cascade of inflammation-mediated tissue damage within the CNS [19]. In addition, B cells contribute to MS pathogenesis through antibody production, antigen presentation, and cytokine release, further amplifying neuroinflammation and lesion formation [19] (Figure 1).

Under normal conditions, the blood–brain barrier (BBB) restricts immune cell entry into the CNS; however, in MS, increased BBB permeability permits the infiltration of activated immune cells, including T lymphocytes and B cells, into the brain and spinal cord [20,21]. These cells recognize myelin antigens as foreign, initiating a localized inflammatory cascade that leads to focal demyelinated lesions, commonly referred to as plaques [22].

Once within the CNS, autoreactive T cells release pro-inflammatory cytokines that amplify immune activation and recruit additional inflammatory cells, including macrophages and microglia [23]. Concurrently, B cells contribute to MS pathogenesis through antigen presentation, cytokine secretion, and the production of autoantibodies against myelin components [24,25]. These antibodies can activate the complement system, further exacerbating myelin injury and axonal damage [22]. Sustained inflammation results not only in demyelination but also in direct axonal injury and neuronal loss, which are increasingly recognized as major drivers of irreversible disability [26,27]. Although some degree of remyelination may occur early in the disease course, repeated inflammatory insults and oligodendrocyte dysfunction ultimately impair repair mechanisms, leading to chronic neurodegeneration [28]. Importantly, accumulating evidence suggests that MS is a heterogeneous disease, with variability in immune mechanisms and degrees of inflammatory versus degenerative pathology across disease subtypes and stages [22].

Beyond its motor and sensory manifestations, MS is frequently associated with a broad range of cognitive, behavioral, and psychiatric symptoms that significantly contribute to disease burden and reduced quality of life [29,30]. Cognitive impairment occurs in approximately 39–64% of individuals with MS and can be present at any stage of the disease, including early and clinically isolated syndromes [31,32]. The most affected cognitive domains include information processing speed, attention, executive function, learning, and memory, while language and general intelligence are relatively preserved [29]. These deficits are thought to arise from a combination of demyelination, axonal injury, cortical and subcortical gray matter damage, and disrupted network connectivity, rather than focal lesions alone [33,34].

Psychiatric manifestations are also highly prevalent in MS and often coexist with cognitive dysfunction. Depression is the most common psychiatric comorbidity, with lifetime prevalence rates substantially higher than those observed in the general population or in other chronic neurological diseases [30,35]. Anxiety disorders, bipolar disorder, and emotional dysregulation, including irritability and affective lability, are also frequently reported [36]. These psychiatric symptoms are not solely reactive to disability or psychosocial stressors; rather, evidence suggests a direct neurobiological contribution related to inflammatory activity, cytokine signaling, and structural and functional changes in limbic and prefrontal regions [37,38]. Neuroinflammation and immune-mediated mechanisms may influence neurotransmitter systems and neural circuits involved in mood regulation, providing a mechanistic link between MS pathology and psychiatric symptoms [39].

Behavioral changes, including apathy, reduced motivation, impulsivity, and social withdrawal, further complicate the clinical presentation of MS and may be independent of depression or physical disability [38,40]. These symptoms are increasingly recognized as manifestations of frontal–subcortical network dysfunction and gray matter involvement. Collectively, cognitive, behavioral, and psychiatric symptoms represent core features of MS rather than secondary consequences, underscoring the need for comprehensive assessment and integrated neuropsychiatric care throughout the disease course [30,35].

## Treatment of Multiple Sclerosis

There are multiple treatment options for differing stages of MS. Disease modifying therapies (DMTs) are a common choice to limit CNS inflammation, slow disability progression, and reduce relapse frequency [41]. DMTs alter the natural development of the MS by targeting immune system mechanisms underlying the disease. Evidence from network meta-analyses indicates that all currently approved DMTs significantly reduce the annualized relapse rate (ARR) compared with placebo, with high-efficacy agents such as alemtuzumab, ocrelizumab, and natalizumab showing particularly strong effects on ARR and disability outcomes [42, 43]. These therapies work by targeting different parts of the immune system to reduce the abnormal autoimmune response that causes MS [41].

Traditional injectable DMTs such as interferon-beta and glatiramer acetate were among the first pharmacotherapies approved for relapsing MS and remain important options, particularly for patients with milder disease activity or specific comorbidity profiles [16,41]. These agents help to regulate the immune system by altering cytokine profiles and shifting T-cell populations toward regulatory phenotypes, which helps decrease inflammatory lesion formation and relapse frequency [23,42]. Although their efficacy is modest compared with newer therapies, these agents established the role of immunomodulation in MS and provided safety data that inform long-term treatment decisions [41,42].

Oral small-molecule DMTs including sphingosine-1-phosphate (S1P) receptor modulators and others such as dimethyl fumarate, teriflunomide, and cladribine have expanded patient choice through convenient dosing and distinct mechanistic profiles [44]. S1P receptor modulators (e.g., fingolimod, ozanimod, siponimod) trap autoreactive lymphocytes in lymphoid tissues, preventing CNS infiltration and reducing disease activity [45]. Newer agents like ozanimod yield significant decreases in clinical relapses compared with interferon-β [45]. Dimethyl fumarate and teriflunomide reduce oxidative stress and lymphocyte proliferation, respectively, and have shown consistent benefits on relapse rate and MRI outcomes across multiple randomized studies [42,43].

Monoclonal antibody therapies targeting specific immune components represent some of the most potent DMTs currently available [43]. B-cell-depleting agents such as ocrelizumab and ofatumumab have shown robust reductions in relapse rate, MRI lesion activity, and disability progression compared with older interferon-based therapies in both relapsing and primary progressive MS, with ocrelizumab demonstrating particularly favorable outcomes in real-world cohort comparisons [43,46]. Observational data further highlight that switching to high-efficacy agents like ocrelizumab after natalizumab cessation is associated with lower relapse rates and longer relapse-free intervals than switching to some oral agents, supporting their use in more refractory cases [47].

Finally, effective MS management increasingly emphasizes personalized treatment selection based on disease activity, progression risk, comorbidities, and patient preferences. While most DMTs primarily target relapses and inflammatory activity, a small number like siponimod have demonstrated benefit in secondary progressive MS populations by slowing confirmed disability progression, and ongoing research continues to explore mechanisms that could impact both relapsing and progressive disease courses [43]. The evolution of therapeutic options underscores the multifaceted nature of MS and the need for evidence-based, individualized care strategies.

## Adherence Issues and Long-term Safety Concern of MS Treatment

Adherence to long-term DMTs in multiple sclerosis remains a significant clinical challenge and can substantially influence both disease progression and treatment effectiveness. Published adherence rates for DMTs vary widely, ranging from approximately 27% to over 88% depending on study design, population, and measurement method, underscoring the inconsistency and suboptimal nature of adherence across the MS population [48]. This variability emerges across different forms of DMTs—including self-injectable, oral, and infusion therapies—highlighting that adherence is a multifactorial issue shaped by treatment modality, patient factors, and disease characteristics [49]. Systematic reviews also reveal that common barriers include adverse events associated with therapy, cognitive impairment, and treatment administration challenges, such as injection-site reactions and dosing complexity, which can negatively impact patients’ ability and willingness to maintain consistent therapy over time [50].

Psychological and behavioral factors also play a crucial role in DMT adherence. Symptoms that are common in MS, such as depression, anxiety, and cognitive dysfunction, have been identified as significant predictors of poorer treatment adherence, likely because they interfere with motivation, routine establishment, and self-management abilities [51]. Moreover, patient perceptions of treatment benefit and risk influence adherence behavior; individuals who believe strongly in the effectiveness of their DMT are more likely to adhere, whereas negative beliefs about side effects and treatment burden correlate with reduced persistence [52]. Poor adherence is not merely a behavioral concern but has measurable clinical consequences: longitudinal real-world analyses demonstrate that non-adherent patients experience higher relapse rates, faster progression to disability milestones, and increased healthcare utilization compared to those with higher adherence [53].

In addition to adherence issues, long-term safety concerns vary substantially across the spectrum of DMTs due to differences in mechanism of action and degree of immune modulation. Traditional platform therapies such as interferon-β and glatiramer acetate have long safety track records, with side effects that are typically mild (e.g., injection-site reactions, flu-like symptoms) and well-characterized over decades of use [54]. In contrast, newer and higher-efficacy agents, particularly those with more profound immunosuppressive effects, carry increased risk profiles that require ongoing attention [55]. For example, therapies that affect lymphocyte trafficking can increase the risk of rare but serious infections like progressive multifocal leukoencephalopathy (PML), especially with longer treatment duration [55]. Anti-CD20 therapies and immune reconstitution therapies such as alemtuzumab can also have long-term safety concerns, including infections and secondary autoimmune conditions, due to their lasting effects on B and T cell populations [55,56].

The combination of adherence challenges and long-term safety concerns complicates clinical decision-making. Patients fearful of adverse effects may be less inclined to remain adherent, even if discontinuation increases the risk of disease reactivation; evidence suggests that cessation or poor adherence to DMTs can lead to subclinical and clinical disease recurrence [57]. Simultaneously, extended exposure to potent immunomodulatory agents necessitates careful monitoring for rare but severe outcomes, particularly as MS patients age and accumulate comorbidities that further heighten vulnerability to treatment-related complications [58]. These dynamics underscore the need for personalized care strategies that balance efficacy, safety, and individual preferences and highlight gaps in managing cognitive and psychiatric symptoms that may contribute to non-adherence in MS.

## Knowledge Gaps in MS Treatment and Support

MS is a chronic autoimmune and neuroinflammatory disease characterized by inflammation, demyelination, and neurodegeneration within the CNS, but crucial aspects of its underlying pathophysiology remain incompletely understood [59]. While abnormal immune responses involving T cells, B cells, cytokines, and disruption of the blood–brain barrier are known to contribute to myelin damage, current treatments mainly focus on reducing peripheral immune inflammation and do not fully stop neurodegeneration or support remyelination [60]. This gap highlights the need for deeper insight into CNS-intrinsic processes, including glial dysregulation, mitochondrial dysfunction, and mechanisms of axonal injury that are not sufficiently addressed by existing therapies [61].

Progressive forms of MS are mainly driven by long-term neurodegeneration rather than the acute immune activity seen in relapsing MS [41]. Because the exact mechanisms behind this ongoing damage are not fully understood yet, there are still limited effective treatment options for progressive disease [41]. Current evidence suggests that innate immune cell activation and compartmentalized inflammation behind an intact blood–brain barrier contribute to ongoing damage, but the precise drivers of these processes are poorly understood [62,63]. This gap limits the development of targeted neuroprotective and remyelination strategies for progressive forms of MS.

Importantly, cognitive impairment, which affects a substantial proportion of people with MS (estimates range from ~39% to over 79% depending on measurement and disease subtype), represents a major, though frequently undervalued, area of disease impact [64,65]. Cognitive deficits commonly involve processing speed, memory, attention, and executive function, and they often develop independently from physical disability, suggesting unique underlying mechanisms such as network dysfunction, gray matter atrophy, and disrupted connectivity across brain regions [66]. However, the neurobiological basis of cognitive impairment remains incompletely mapped, and there is insufficient evidence regarding how different disease processes, notably lesion burden versus diffuse neurodegeneration, each contribute to cognitive decline [67].

Psychiatric symptoms, particularly depression and anxiety, are also highly prevalent in MS and frequently co-occur with cognitive deficits, suggesting shared or interacting neurobiological pathways [68]. Yet, clinical care often underrecognizes and undertreats these symptoms, and research has not fully elucidated how inflammatory mediators and neuroanatomical changes contribute to psychiatric comorbidity in MS [69]. The interplay between mood disorders and cognitive dysfunction in MS is particularly understudied despite evidence that depression can exacerbate cognitive impairment and reduce quality of life [70].

Another important gap is how basic science findings are applied to patient care. In practice, things like cognitive function, mood, and psychosocial challenges are not always regularly assessed, and there is still a lack of well-developed interdisciplinary approaches that address these issues alongside physical disability [71,72]. This suggests a disconnect between epidemiological evidence showing high prevalence of cognitive and mental health concerns and the clinical prioritization of these issues, in part because interventions specifically targeting cognitive and psychiatric outcomes are limited and lack robust evidence from controlled trials [73].

Finally, current research highlights the need for more targeted therapeutic strategies that extend beyond immunomodulation, including those aimed at promoting remyelination, protecting neurons from degeneration, and directly addressing cognitive and psychiatric symptoms [74,75]. Without such advances, treatments will continue to reduce relapse rates and MRI activity but remain insufficient in preventing long-term disability progression, particularly in domains most closely tied to quality of life [74].

## Autoimmune Encephalitis: An Overview and Underlying Mechanisms

**Figure 2:**
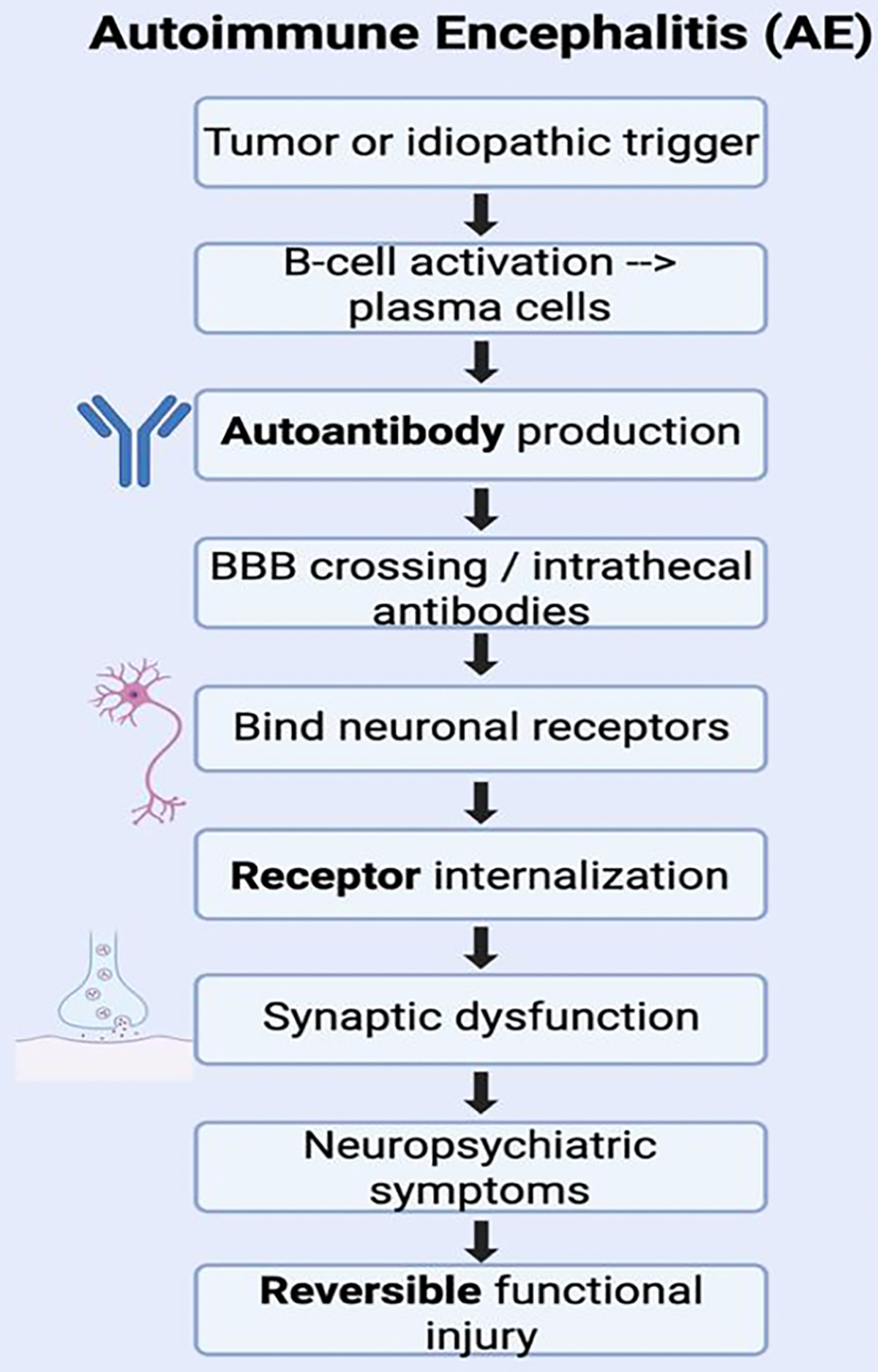
Pathophysiological Mechanisms of Autoimmune Encephalitis. Autoimmune responses are triggered with the production of autoantibodies that cross the blood-brain barrier (BBB), bind to neuronal receptors and induce synaptic dysfunction. This results in neuropsychiatric symptoms.

Autoimmune encephalitis (AE) is an inflammation of brain parenchyma attributed to autoantibodies targeting specific synaptic cell surface structures such as receptors, ion channels and surface proteins or intracellular epitopes, with its spectrum expanding due to the recognition of a myriad of new antibodies [76]. The diverse clinical presentation of this disorder imposes a barrier to its recognition, but recent clinical definitions have been aiding in its early diagnosis and prompt treatment [76]. The pathophysiology of this disorder is dependent on its specific target as well as the location of the target. Antibodies targeting cell-surface structures are differentiated from those targeting intracellular antigens due to their distinct mechanism [76,77] (Figure 2).

In addition to antibodies affecting synaptic function, autoimmune encephalitis also involves more widespread immune activation that disrupts normal brain function and balance [77]. Inflammatory cytokines, microglial activation, and alterations in blood–brain barrier permeability all contribute to changes in neural network function [78]. These processes can affect neurotransmitter systems, including glutamatergic, GABAergic, and dopaminergic pathways, which are critical for regulating cognition, mood, and behavior [79]. The specific clinical presentation of AE often depends on the target antigen and the brain regions involved. For example, limbic system involvement is associated with memory impairment, emotional dysregulation, and seizures, while cortical and subcortical involvement may lead to psychosis, movement disorders, or altered consciousness [80]. Because these immune-mediated disruptions primarily affect functional signaling rather than causing immediate structural damage, symptoms can fluctuate and evolve over time, often progressing from subtle psychiatric changes to more severe neurological dysfunction [76,80]. This variability in presentation further contributes to diagnostic challenges and highlights the importance of understanding AE as a disorder of both immune dysregulation and network-level brain dysfunction (Figure 2).

AE presents with a wide range of neurological and psychiatric symptoms, which contributes to frequent delays in diagnosis [81]. Early in the disease course, patients often develop psychiatric symptoms such as anxiety, agitation, paranoia, or hallucinations, sometimes without obvious neurological deficits [82]. This is especially well described in anti-NMDA receptor encephalitis, where psychiatric symptoms can precede seizures, movement disorders, or autonomic instability [81,82]. Because of this, patients are commonly misdiagnosed with primary psychiatric conditions such as schizophrenia, bipolar disorder, or substance-induced psychosis [83]. Additional challenges include nonspecific MRI findings, variability in cerebrospinal fluid (CSF) abnormalities, and delays in antibody testing, all of which can slow recognition of the disease [84]. Literature consistently emphasizes that delayed diagnosis is associated with worse outcomes, highlighting the importance of early clinical suspicion and prompt initiation of treatment [82,84].

Psychiatric and cognitive symptoms are not only common but often central to the presentation of AE. These can include psychosis, mood disturbances, irritability, memory impairment, and executive dysfunction [85]. In more severe cases, patients may develop catatonia, decreased level of consciousness, or significant behavioral dysregulation [86]. Studies have shown that even after resolution of the acute inflammatory phase, many patients continue to experience long-term cognitive deficits, particularly in memory and attention, as well as persistent depression and anxiety [87]. These findings support the idea that AE affects large-scale neural networks involved in cognition and emotion, and that recovery of neurological function does not always translate to full neuropsychiatric recovery [77,87].

## Treatment Strategies for AE

Treatment of autoimmune encephalitis is centered on early and aggressive immunotherapy to reduce immune-mediated neuronal dysfunction and improve clinical outcomes [76]. First-line therapies typically include high-dose corticosteroids, intravenous immunoglobulin (IVIG), and plasmapheresis [88]. These treatments aim to reduce inflammation, modulate immune signaling, and remove circulating pathogenic antibodies [88,89]. In many cases, a combination of these therapies is used depending on disease severity and clinical response. If patients do not improve with first-line therapy, second-line treatments such as rituximab, a monoclonal antibody targeting CD20+ B cells, or cyclophosphamide may be initiated to further suppress the immune response [89,90]. In paraneoplastic cases, identification and treatment of the underlying tumor is also essential, as tumor removal can significantly improve neurological outcomes [76].

The effectiveness of treatment is strongly dependent on early recognition and initiation of therapy. Multiple observational studies have shown that patients treated earlier in the disease course are more likely to have favorable outcomes and functional recovery [91]. Many patients experience rapid improvement in acute symptoms such as psychosis, seizures, and altered mental status following immunotherapy [76,88]. However, recovery is often prolonged and may occur over months to years. Importantly, even in patients who achieve good functional outcomes based on standard neurological scales, a significant proportion continue to experience persistent cognitive and psychiatric symptoms [92]. These may include deficits in memory, attention, and executive function, as well as ongoing depression, anxiety, or fatigue [93]. This discrepancy highlights a limitation in commonly used outcome measures, which may underestimate the true burden of disease by focusing primarily on physical or motor recovery [94].

Despite advances in treatment, several important knowledge gaps remain [95]. Much of the current evidence guiding AE management is based on observational studies and expert consensus rather than randomized controlled trials, limiting the ability to establish standardized treatment protocols [96]. There is also limited research on long-term management of cognitive and psychiatric symptoms, and structured rehabilitation strategies are not well defined for this population. Additionally, reliable biomarkers to predict treatment response, disease severity, and long-term outcomes are still lacking [94]. Future research should focus on improving early diagnostic tools, developing targeted immunotherapies, and integrating neuropsychiatric care into standard treatment approaches to better address the full spectrum of disease burden.

## Biological vs. Psychosocial Drivers of Chronic Inflammation

The psychological impact of AE and MS cannot be adequately explained by either inflammatory biology or psychosocial stressors alone. Contemporary literature in neuroimmunology and psychoneuroimmunology supports a bidirectional model in which immune activation, CNS network disruption, and chronic stress physiology interact to shape mood, cognition, and behavior [97,98]. In both AE and MS, psychiatric and cognitive symptoms arise from intertwined biological mechanisms such as cytokine signaling, microglial activation, and hypothalamic–pituitary–adrenal (HPA) axis dysregulation, while simultaneously being amplified or sustained by psychosocial drivers including illness uncertainty, disability, stigma, and trauma [99,100]. Framing these conditions through a dual biological–psychosocial lens provides a more accurate account of patient experience and highlights why purely neurologic disease control often fails to fully restore cognitive and emotional functioning [101,102].

A growing body of peer-reviewed research demonstrates that peripheral and central immune activation can directly influence neural circuits involved in mood, motivation, and cognition [97,103]. Inflammatory cytokines communicate with the brain through multiple routes, including altered blood–brain barrier permeability, endothelial signaling, vagal afferent pathways, and, under specific conditions, trafficking of immune cells into the CNS [79]. These signals shift microglial and astrocytic states and alter synaptic plasticity, neurotransmitter systems, and network connectivity [104]. Major publications in journals such as Nature Reviews Immunology and Molecular Psychiatry have described how inflammatory signaling can affect monoaminergic transmission, glutamatergic balance, and hippocampal neurogenesis, offering biologically plausible mechanisms for depression, fatigue, slowed processing speed, and executive dysfunction in chronic inflammatory diseases [80,97]. These mechanisms are particularly relevant in MS, where diffuse neuroinflammation and neurodegeneration compromise white matter tracts and large-scale networks, and in AE, where immune processes may directly target synaptic receptors and limbic circuits that govern behavior and affect [77,99].

HPA axis dysregulation further bridges inflammation and psychiatric symptomatology [105,106]. Acute stress responses may transiently modulate immune activity, but chronic stress can alter glucocorticoid signaling and, in some contexts, lead to relative glucocorticoid resistance, allowing inflammatory pathways to remain active despite elevated cortisol levels [107,108]. Reviews of stress immunology consistently demonstrate that sustained psychosocial stress reshapes immune cell distribution and cytokine profiles through both HPA and sympathetic pathways [109,110]. In chronic neurologic disease, this creates a reinforcing loop in which stress related to symptoms or disability perpetuates inflammatory signaling, which in turn exacerbates mood and cognitive disturbances [97,100]. The kynurenine pathway of tryptophan metabolism provides another mechanistic link; inflammatory activation shifts tryptophan metabolism toward neuroactive metabolites that interact with glutamatergic systems, potentially contributing to cognitive dysfunction and depressive symptoms [80,103]. Together, these pathways illustrate how inflammation is not merely correlated with psychiatric symptoms but may actively participate in their pathophysiology [97].

Reviews in neuropsychiatric journals emphasize that disorders such as anti–NMDA receptor encephalitis frequently present with psychiatric changes, often preceding obvious neurologic deficits [81,85]. Although immunotherapy can produce substantial clinical improvement, longitudinal outcome studies published in neurology journals demonstrate that many survivors experience persistent cognitive impairments [87,93]. Importantly, traditional functional outcome measures such as the modified Rankin Scale often underestimate this burden, because patients may regain motor independence while continuing to struggle with neuropsychological deficits that impair employment, academic performance, and social reintegration [94,111]. This discrepancy highlights a significant gap between neurologic recovery and true functional recovery [111].

In MS, psychiatric comorbidity is among the most common and disabling nonmotor manifestations of the disease [41,43]. Depression and anxiety prevalence rates exceed those of the general population, and these symptoms meaningfully influence quality of life, treatment adherence, and disability progression [42]. Contemporary reviews underscore that MS-related depression is not purely due to the stress of being diagnosed with disease; inflammatory signaling, lesion distribution, cortical and subcortical atrophy, fatigue, sleep disruption, and pain all contribute biologically [43,46]. Overall, the evidence suggests that both inflammation and psychosocial factors work together to affect the same brain circuits that control mood and thinking [103].

Psychosocial drivers deserve equal emphasis because chronic neurologic illness reshapes identity and daily functioning. Patients with AE often endure prolonged hospitalizations, intensive care stays, behavioral dysregulation, and periods of amnesia, experiences that can be traumatic for both patients and caregivers [85,112]. Survey-based and longitudinal studies of encephalitis survivors document high rates of anxiety, sleep disturbance, and mood disorders years after the acute event [93,94]. In MS, invisible symptoms such as fatigue and slowed processing speed frequently lead to misunderstanding or minimization by employers and even clinicians, compounding distress and social isolation [32,35]. Chronic stress from these situations has real biological effects, as it activates stress hormone pathways that can impact the immune system [103]. So, psychosocial stress is not just separate from the disease but can interact with inflammatory processes to worsen and maintain symptoms [46].

**Figure 3:**
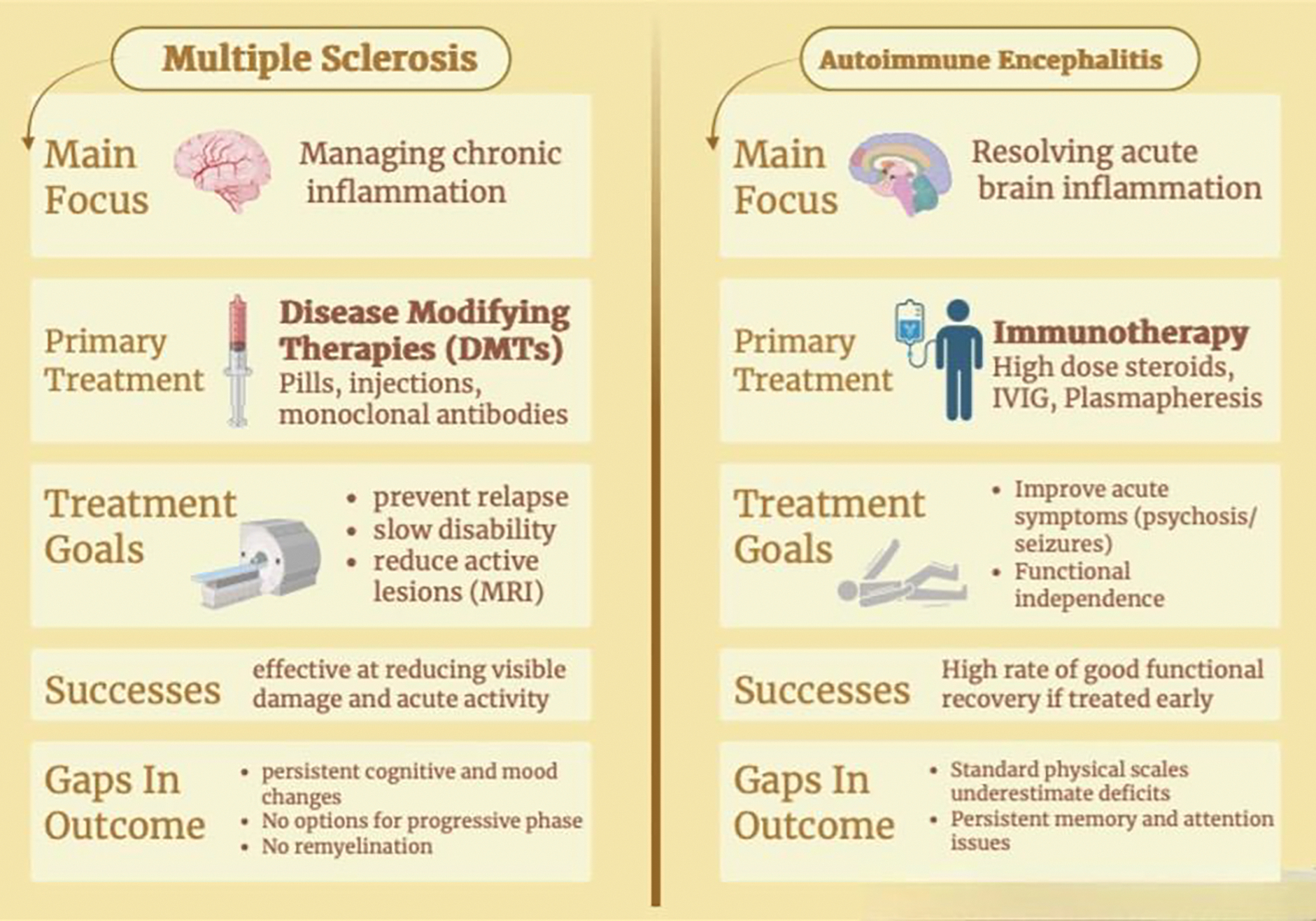
Therapeutic Strategies and Clinical Outcomes in Multiple Sclerosis and Autoimmune Encephalitis.

Given this complex interplay, treatment approaches that target inflammation alone are insufficient to address cognitive and psychiatric sequelae [46,111]. Early and effective immunotherapy remains foundational in AE, particularly because psychiatric manifestations may reflect direct autoimmune synaptic disruption [85]. In MS, disease-modifying therapies reduce inflammatory activity and relapse frequency, but mood and cognitive symptoms often require dedicated parallel interventions [43,46]. The literature consistently supports embedding structured psychiatric screening and neuropsychological assessment into routine neurologic follow-up, as reliance on global disability scales obscures substantial cognitive and emotional morbidity [87,94]. Yet implementation remains inconsistent, representing a major systems-level gap in care (Figure 3).

Multidisciplinary treatment models offer the most coherent response to the dual biological and psychosocial drivers of symptom burden (Figure 3). Psychiatric care, including evidence-based pharmacotherapy and psychotherapy, addresses mood and anxiety disorders while considering disease-specific factors such as corticosteroid exposure or sleep disruption [43,46,113]. Neuropsychological evaluation helps identify specific cognitive deficits and allows for more tailored rehabilitation plans [94]. Occupational therapy, speech-language pathology, and cognitive rehabilitation programs provide compensatory techniques and targeted cognitive training [114]. In MS, randomized controlled trials demonstrate that tailored psychological interventions can significantly reduce depressive symptoms, highlighting the feasibility of scalable, disease-specific mental health care [115,116]. Meta-analyses of rehabilitation approaches, including virtual reality–based and computerized cognitive training, show promising improvements in cognitive domains, though effects on mood are more variable and methodological heterogeneity persists [117,118].

In AE, cognitive recovery may continue for years, yet standardized rehabilitation pathways remain underdeveloped compared with MS [87]. Outcome studies reveal that even patients classified as functionally independent frequently report persistent memory deficits, executive dysfunction, and psychiatric symptoms [87,93]. Controlled trials of structured cognitive rehabilitation in AE are limited, and best practices are often estimated from broader neurorehabilitation literature [119]. This gap underscores the need for prospective, disease-specific studies that integrate neuropsychological endpoints, patient-reported outcomes, and long-term psychiatric follow-up [94].

Overall, contemporary literature supports a model in which chronic inflammation exerts both direct neurobiological effects on mood and cognition and indirect effects through psychosocial stress and identity disruption [46]. Treatment successes include earlier recognition of autoimmune neuropsychiatric syndromes, improved immunotherapy protocols, and growing evidence for psychological and cognitive rehabilitation interventions in MS [41,76,85]. However, important gaps still exist, including inconsistent long-term screening, under-recognition of ongoing psychiatric symptoms despite neurological recovery, a lack of AE-specific rehabilitation studies, and limited integration of mental health services within neuroimmunology care [72,93,94]. Addressing these gaps requires looking beyond just lesion-based models and instead using a more integrated biopsychosocial approach, where cognitive and psychiatric symptoms are recognized as central parts of the disease rather than just secondary complications.

## Future Direction and Research Needs

The literature on autoimmune encephalitis and multiple sclerosis clearly illustrates that while significant progress has been made in understanding and treating the neurological aspects of these diseases, the psychological and cognitive presentations remain under‐addressed [68,94]. As a result, future research must prioritize bridging the gaps between peripheral and central immune activity, neural network dysfunction, and psychiatric/cognitive symptoms through more precise biomarkers, better designed clinical trials, and deeper mechanistic studies [23,84,103]. A recurring challenge in current work is that studies often treat psychiatric outcomes as secondary, rather than core outcomes of disease activity [71,94]. This limits the ability to understand causal pathways and to design interventions that effectively prevent or reverse mood, anxiety, and cognitive sequelae in inflammatory neurologic diseases [68,93].

One promising future direction is developing biomarkers that link inflammation to psychiatric and cognitive symptoms in AE and MS [84,103]. While markers like cytokines, acute-phase proteins, and neuroendocrine signals are associated with depression and anxiety in general populations, their role in these diseases is still unclear [68,84]. Ideally, biomarkers could distinguish immune or genetic signals that predict persistent psychiatric symptoms from those reflecting general inflammation [23,84]. For example, cytokines such as IL-6 and TNF-α have been linked to depression across autoimmune disorders and may serve as useful candidates [72]. Markers of blood–brain barrier damage, like CSF/serum albumin ratios or neurofilament light chain, may also help show how central inflammation contributes to psychiatric outcomes [121,122]. Longitudinal studies tracking immune activity, neural injury, and psychiatric symptoms together could clarify prediction of progression and treatment response [84,93].

Another key area is improving clinical trials by including mental health outcomes and longer follow-up periods [71,72]. Many AE and MS trials focus mainly on neurological measures, while psychiatric outcomes are often secondary or short-term [71,94]. These limits understanding of long-term depression, anxiety, and cognitive impairment [68,93]. Future trials should include standardized psychiatric and cognitive assessments as primary or co-primary outcomes [71,93]. They should also follow patients longer, since psychiatric symptoms may appear or persist years after treatment [87,93]. Longer follow-up would also help evaluate long-term treatment effects and supportive therapies like CBT and rehabilitation [68,71].

Finally, mechanistic studies linking immune activity to brain function are important [103]. Combining imaging, CSF analysis, and brain activity measures could show how immune changes affect mood and cognition circuits [23,84]. For example, multimodal imaging may reveal patterns of brain disruption tied to inflammation [23,125]. Studies of glutamate signaling and synaptic changes could further explain cognitive and motivational symptoms [103]. Integrating animal and human research may help identify causal pathways and new treatment targets [103].

Another important area of research is genetics and gene expression. Studies in broader psychiatric populations have found immune-related genetic risk factors for depression and anxiety, and applying similar approaches in AE and MS could help reveal how inherited factors influence interactions between the immune system and the brain [103,126]. Single-cell transcriptomics of blood and CSF immune cells, paired with neural tissues, when possible, might reveal cell subtypes or activation states that are particularly relevant to psychiatric symptom expression [84,127]. Combining these data with clinical characteristics could help researchers identify molecular patterns that not only indicate risk but also point toward potential targeted treatments [84,68].

In addition to these core research priorities, broader systemic improvements will be necessary. Using consistent cognitive and psychiatric measures across studies will make it easier to compare results and combine findings from different trials and observational studies [93,94]. Collaborative groups that combine data from multiple centers and countries can create larger sample sizes, making it easier to detect meaningful effects and confirm important biomarkers or findings [23]. Funding agencies and research institutions must recognize the critical importance of mental health outcomes in immune-mediated neurologic disease, elevating these outcomes equally with classic neurologic endpoints in grant review criteria and clinical practice guidelines [71,72].

In summary, future research in the field of neuroimmune disorders must pivot toward integrative models that treat psychiatric and cognitive outcomes as central to the disease processes. Biomarkers that link inflammation and psychiatric symptoms, clinical trials with integrated and longitudinal mental health outcomes, and mechanistic studies that bring to light the immune-to-brain pathways are all urgently needed [71,84,103]. Advances in these areas will not only deepen scientific understanding but also inform more holistic, effective care models that address both neurological and psychological recovery, ultimately improving long-term functioning and quality of life for patients with AE, MS, and related disorders [68,93].

### Potential Limitations

**Figure 4:**
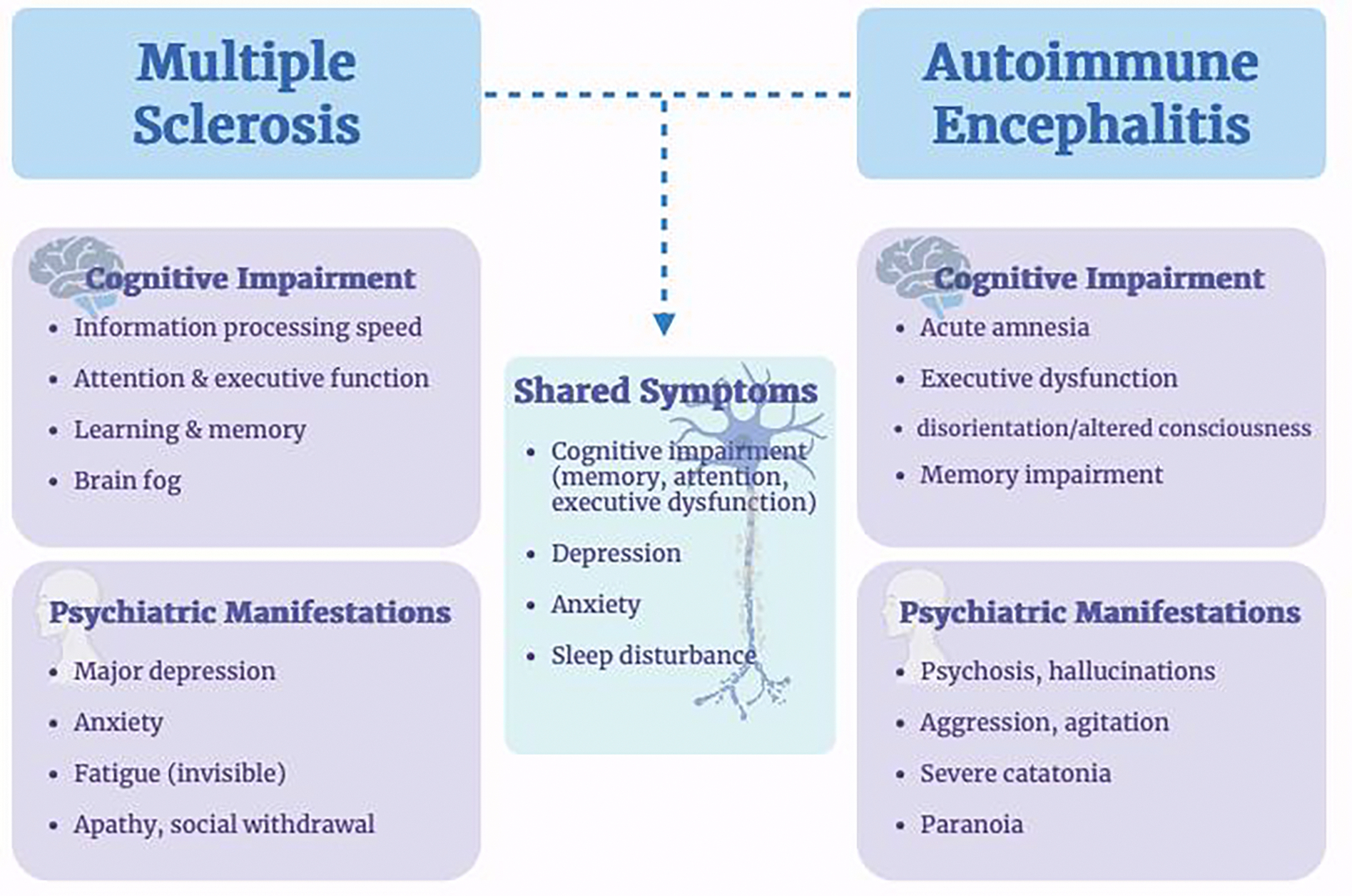
Neuropsychiatric, Cognitive Manifestations, and Shared Symptoms in Multiple Sclerosis and Autoimmune Encephalitis.

Current evidence demonstrates that cognitive and psychiatric symptoms in AE and MS are clinically significant and interestingly there are several shared symptoms (Figure 4). However, several methodological limitations limit translation into consistent, evidence-based care [68,94]. A major issue across both conditions is heterogeneity in study design, including differences in patient populations, outcome measures, and timing of assessments, which makes it difficult to compare results across studies [93,94]. In AE, outcomes are often measured using global disability scales such as the modified Rankin Scale, which may classify patients as having good recovery despite persistent cognitive and psychiatric deficits. This may lead to underestimation of the true burden of disease [93,94].

In AE specifically, this limitation is further complicated by the biological and clinical diversity of the disease. Different antibody subtypes are associated with distinct clinical presentations, disease severity, and long-term outcomes, yet many studies group these subtypes together or use inconsistent diagnostic criteria [76,85]. In addition, most evidence guiding treatment and outcomes is derived from observational studies rather than randomized controlled trials, making it difficult to establish standardized management strategies or determine causality [94]. Even in patients who are in recovery, persistent cognitive and psychiatric symptoms are frequently reported, highlighting a gap between traditional neurological recovery measures and patient-reported outcomes [87,93].

Another important limitation is the lack of robust longitudinal data, particularly for cognitive and psychiatric outcomes [93,94]. Much of the current literature relies on cross-sectional or retrospective studies with small sample sizes, which limits the ability to track symptom progression over time [93]. In both AE and MS, psychiatric symptoms such as depression and anxiety are common but inconsistently measured, and cognitive impairment is often underrecognized or assessed using non-standardized tools [68,71,72]. Additionally, short follow-up periods in many studies fail to capture the prolonged and often nonlinear recovery seen in these conditions, particularly in AE where recovery may continue over several years [87,93].

Similarly in MS, although some standardized tools for cognitive assessment exist, variability in measurement methods and study design continues to limit comparability across studies [60,82]. Furthermore, overlap between cognitive symptoms, fatigue, and mood disorders can complicate interpretation of results if not carefully accounted for in study design and analysis [46,68].

Finally, many studies in both AE and MS are limited by small sample sizes and insufficient power to detect long-term cognitive and psychiatric outcomes, particularly when analyzing subgroups [93,94]. This is especially true in AE due to its relative rarity [85]. As a result, there remains uncertainty regarding the prevalence, progression, and treatment responsiveness of neuropsychiatric symptoms [68,93]. Future research should focus on developing standardized outcome measures, incorporating longitudinal follow-up, and conducting larger, multicenter studies to better define symptom trajectories and improve long-term management [71,94]. These improvements are important for closing the gap between visible neurological recovery and ongoing cognitive and psychiatric symptoms [87,94].

## Conclusions

Neuroinflammatory disorders such as multiple sclerosis and autoimmune encephalitis demonstrate how dysregulated immune activity can lead to both neurological and psychiatric symptoms through different but overlapping mechanisms. While multiple sclerosis is characterized by chronic demyelination and neurodegeneration, autoimmune encephalitis more often presents with acute antibody-mediated disruption of neuronal signaling. Despite these differences, both conditions emphasize that immune dysfunction plays a central role in affecting brain function and patient outcomes.

Although current treatments have improved disease control, they are primarily focused on suppressing immune activity and do not always address persistent cognitive and psychiatric symptoms. Evidence suggests that these symptoms are driven not only by inflammation and neural injury, but also by psychosocial factors related to chronic illness. This highlights an important gap in care, as patients may continue to experience significant impairment even when their disease is considered medically stable.

Future research should aim to better integrate biological and psychosocial perspectives by incorporating cognitive and mental health outcomes into clinical studies. A more comprehensive approach that includes biomarkers, long-term follow-up, and multidisciplinary care may help improve overall quality of life for patients with these conditions.

## Key Points

- Multiple sclerosis and autoimmune encephalitis are neuroinflammatory diseases that produce both neurological and psychiatric symptoms through immune-mediated mechanisms
- Cognitive impairment and psychiatric presentations are common in both conditions and significantly impact quality of life
- In multiple sclerosis, symptoms arise from a combination of demyelination, axonal injury, and disrupted neural network connectivity
- In autoimmune encephalitis, antibody-mediated synaptic dysfunction and widespread immune activation disrupt neural signaling and behavior
- Current treatments primarily target physical inflammation but often fail to fully address the persistent cognitive and psychiatric symptoms
- Psychiatric and cognitive outcomes are frequently underrecognized and underassessed in routine clinical care
- Evidence supports an interdependent relationship between immune activity and neural circuits involved in mood, cognition, and behavior
- Psychosocial factors such as chronic stress, disability, and illness uncertainty interact with biological mechanisms to sustain symptom burden
- Multidisciplinary care, including psychiatric treatment and cognitive rehabilitation, is essential for improving long-term outcomes
- Future research should prioritize biomarkers, longitudinal studies, and clinical trials that incorporate cognitive and psychiatric symptoms centrally
